# Synthesis and Biological Evaluation of Bicalutamide Analogues for the Potential Treatment of Prostate Cancer

**DOI:** 10.3390/molecules26010056

**Published:** 2020-12-24

**Authors:** Sahar B. Kandil, Christopher McGuigan, Andrew D. Westwell

**Affiliations:** School of Pharmacy & Pharmaceutical Sciences, Cardiff University, Wales CF10 3NB, UK; McGuigan@cardiff.ac.uk (C.M.); WestwellA@cardiff.ac.uk (A.D.W.)

**Keywords:** androgen receptor (AR), prostate cancer (PC), sulfone, diarylpropionamide, bicalutamide

## Abstract

The androgen receptor (AR) is a pivotal target for the treatment of prostate cancer (PC) even when the disease progresses toward androgen-independent or castration-resistant forms. In this study, a series of 15 bicalutamide analogues (sulfide, deshydroxy, sulfone, and *O*-acetylated) were prepared and their antiproliferative activity evaluated against four different human prostate cancer cell lines (22Rv1, DU-145, LNCaP, and VCap). Bicalutamide and enzalutamide were used as positive controls. Seven of these compounds displayed remarkable enhancement in anticancer activity across the four PC cell lines. The deshydroxy analogue (**16**) was the most active compound with IC_50_ = 6.59–10.86 µM. Molecular modeling offers a plausible explanation of the higher activity of the sulfide analogues compared to their sulfone counterparts.

## 1. Introduction

**Figure 1:**
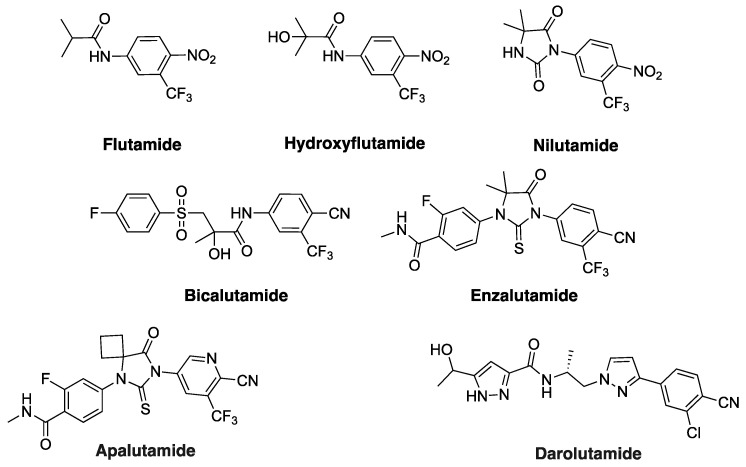
Chemical structures of the nonsteroidal antiandrogens (NSAA); flutamide, hydroxyflutamide, nilutamide, bicalutamide, enzalutamide, apalutamide, and darolutamide.

The androgen receptor (AR) has essential anabolic and reproductive roles in men and women. Additionally, AR signaling plays a crucial function in tumorigenesis and metastasis of different cancer types, including prostate, bladder, kidney, lung, breast, and liver [1,2,3]. AR is a member of the nuclear receptor family and consists of three main functional domains: a variable N-terminal domain, a highly conserved DNA-binding domain (DBD), and a conserved ligand-binding domain (LBD) [4]. Binding of testosterone and dihydrotestosterone (DHT) to the LBD induces AR conformational changes then translocation into the nucleus to interact with DNA and modulate prostate-specific antigen (PSA) levels [5]. AR antagonists (antiandrogens) inhibit these processes and are used for the treatment of advanced prostate cancer (PC) [6,7]. A variety of nonsteroidal antiandrogens (NSAA) are approved for the treatment of PC. The first generation NSAAs include flutamide, hydroxyflutamide, and bicalutamide (Figure 1). However, these antiandrogens eventually fail to inhibit the AR upon long-term treatment, switching from being AR antagonists to AR agonists with the development of castration-resistant prostate cancer (CRPC), an aggressive form of the disease with poor prognosis. Similarly, resistance to the more recent second-generation antiandrogens (enzalutamide, apalutamide) is developing in PC patients via the upregulation of AR expression [8]. More recently, darolutamide (ODM-201) has been recently approved and clinically used in patients with nonmetastatic CRPC [9]. New AR antagonists are continuously needed to improve the efficacy of the clinically used compounds.

In this paper, we present the design and synthesis of a series of new bicalutamide analogues, building on our previous work [10,11,12,13] to offer new therapeutic options for combating the resistance observed in the clinical use of AR antagonists.

## 2. Results and Discussion

Generally, minor chemical changes in the chemical structure of nonsteroidal AR ligands can result in major pharmacological outcomes [14,15]. Previously, we published extensive SAR studies on bicalutamide analogues [10,11,12,13]. Introduction of fluorinated groups into the chemical structure of molecules provides a combination of electrostatic, steric and lipophilic impacts on the physicochemical properties and biological activities [16,17,18].

**Figure 2:**
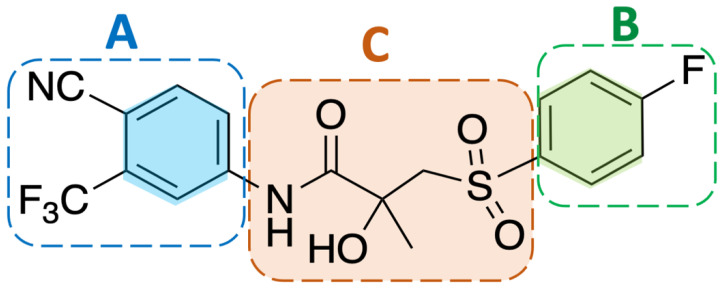
Chemical structure of bicalutamide and the areas of structural modifications, ring (**A**), ring (**B**), and the linker area (**C**).

Here we are exploring the impact of additional chemical structure modifications on the antiproliferative activity in prostate cancer cell lines. The three main areas of modification are ring **A**, ring **B**, and linker area **C** as illustrated in Figure 2. Fifteen bicalutamide derivatives were prepared and their antiproliferative activity was evaluated in vitro against four different human prostate cancer cell lines (22Rv1, DU-145, LNCaP, and VCap).

### 2.1. Sulfide Analogues of Bicalutamide

**Scheme 1:**
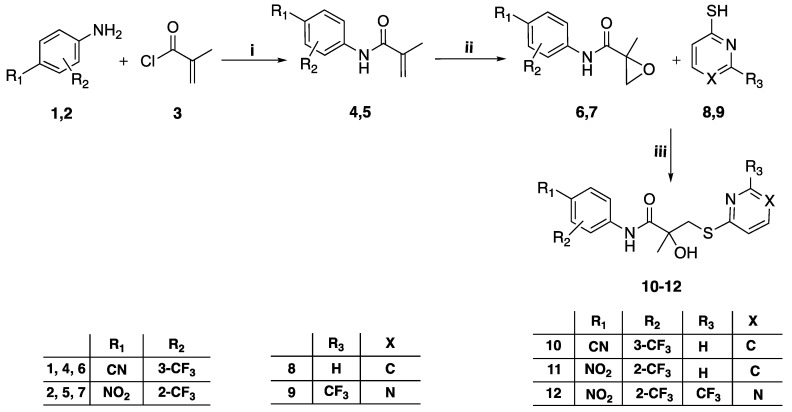
Reagents and conditions, (**i**) DMA, rt, 3 h; (**ii**) H_2_O_2_, TFAA, DCM, rt, 24 h; (**iii**) NaH, THF, RT, 24 h.

Phenylacrylamides (**4** and **5**) were prepared by reacting the corresponding aniline (**1** or **2**) with methacryloyl chloride (**3**) in dimethylacetamide (DMA) [19]. Phenylacrylamides (**4, 5**) were epoxidized by hydrogen peroxide and trifluoroacetic anhydride (TFAA) in dichloromethane to give (**6** and **7**) [20]. Subsequently, the epoxides were reacted with the aromatic thiols (**8** and **9**) to afford the sulfide derivatives (**10**–**12**) as racemic mixtures, as outlined in Scheme 1. 

The sulfide bicalutamide derivatives (**10**–**12**) were evaluated for their antiproliferative activity in vitro against four human prostate cancer cell lines (22Rv1, DU-145, LNCaP, and VCap) using the Oncotest monolayer assay (see Experimental Section). Bicalutamide and enzalutamide were used as positive controls. Compound **12** showed approximately twofold activity improvement in three cell lines, 22Rv1, LNCaP, and VCap (IC_50_ = 16.88−27.30 µM), compared to bicalutamide. Meanwhile, compound **10** displayed enhanced activity (IC_50_ = 23.51–27.94 µM) in 22Rv1 and LNCaP, whereas compound **11** was inactive (> 100 µM) in all cell lines except LNCaP (IC_50_ = 16.34 µM) (Table 1).

### 2.2. Deshydroxy Sulfide Analogues of Bicalutamide

**Scheme 2:**
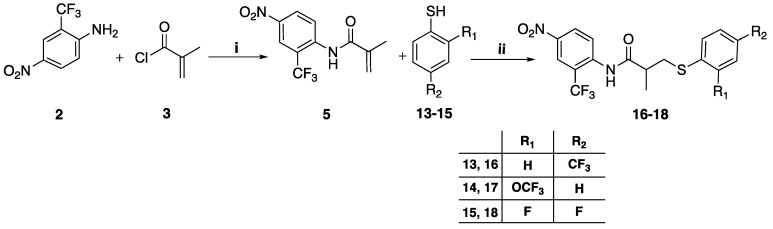
Reagents and conditions, (**i**) DMA, rt, 3 h; (**ii**) NaH, THF, RT, 24 h.

Reacting 4-nitro-2-(trifluoromethyl) aniline (**2**) with methacryloyl chloride (**3**) in dimethylacetamide (DMA) generated phenylacrylamide derivative (**5**) [16], which was subsequently reacted with the appropriate thiols (**13**–**15**) to afford the corresponding deshydroxy sulfide derivatives (**16**–**18**) as racemic mixtures, as described in Scheme 2.

The deshydroxy sulfide bicalutamide derivatives (**16**–**18**) were assessed for their antiproliferative activity in four human prostate cancer cell lines (22Rv1, DU-145, LNCaP, and VCap). Compound **16** displayed approximately 7-fold enhanced activity (IC_50_ = 6.59 µM) in 22Rv1 and 4- to 5-fold enhanced activity (IC_50_ = 8.22−10.86 µM) in DU-145, LNCaP, and VCap, compared to bicalutamide. Similarly, compound **17** showed activity improvement in all cell lines (IC_50_ = 9.99−30.85 µM). However, compound **18** showed loss of activity (> 100 µM) in three cell lines: 22Rv1, DU-145, and VCap, Table 2.

### 2.3. Sulfone Analogues of Bicalutamide

**Scheme 3:**
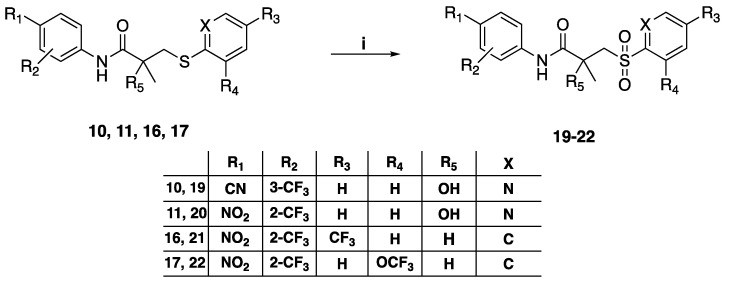
Reagents and conditions, (**i**) *m*CPBA (2 equiv), DCM, rt, 24 h.

Bicalutamide sulfone (SO_2_) derivatives (**19**–**22**) were prepared by oxidation of the corresponding sulfide derivatives using two equivalents of *m*-chloroperbenzoic acid (*m*CPBA) over a reaction time of 24 h at room temperature, resulting in the complete oxidation into sulfone analogues [19], as outlined in Scheme 3.

**Figure 3:**
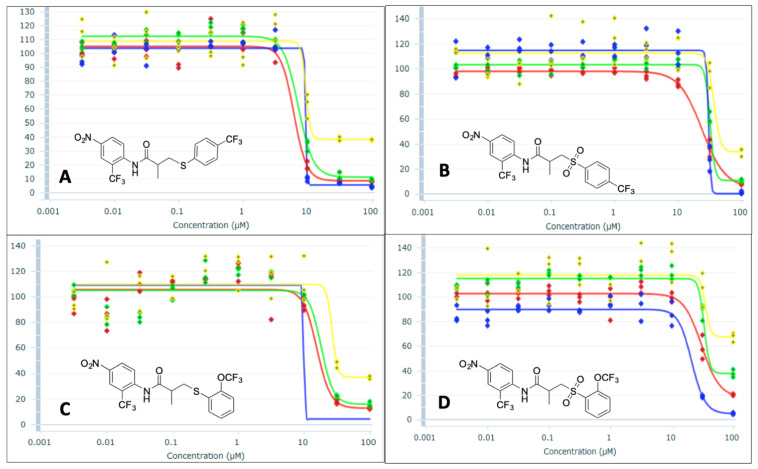
Dose response curves of sulfide compounds **16** (**A**) and **17** (**C**) compared to their corresponding sulfone derivatives **21** (**B**) and **22** (**D**), respectively, in the Oncotest monolayer assay of 22Rv1 (red), DU-145 (green), LnCaP (blue), and VCap (yellow) cell lines.

Evaluation of the antiproliferative activity of the sulfone compounds showed that compounds **21** and **22** retained good activity in the four human prostate cancer cell lines (22Rv1, DU-145, LNCaP, and VCap) but to a lesser extent compared to their corresponding thioether analogues **16** and **17**, respectively, Figure 3. On the other hand, compounds **19** and **20** did not show any activity up to 100 µM, Table 3.

**Figure 4:**
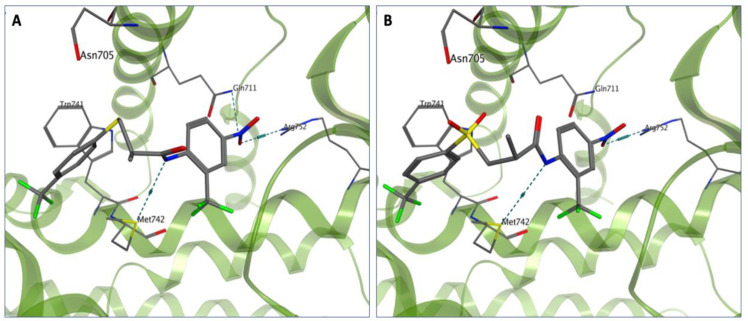
The predicted binding mode of compounds **16** (**A**) and **21** (**B**) within the hAR-LBD showing H-bond interactions (blue dashed line) with Arg 752, Gln 711, Met 742.

An in silico docking study was performed to compare the putative binding modes of the sulfide (S) compound **16** (IC_50_ = 6.59–10.86 µM) and its sulfone (SO_2_) analogue **21** (IC_50_ = 24.64–43.04 µM). Both compounds share key interactions including an H-bond between the nitro group (NO_2_) and the guanidine group of Arg 752 of helix 5. Another H-bond was observed between the amide (NH) and the side chain S-methyl thioether (SCH_3_) group of Met 742 amino acid residue. However, only compound **16** shows an H-bond between its nitro group (NO_2_) and the side chain amide (NH_2_) group of Gln 711 (Figure 4A), which may explain its higher activity compared to its sulfone analogue (**21**) which lacks such interaction (Figure 4B). In addition, hydrophobic interactions were observed between the 4-trifluoromethyl phenyl moiety and the surrounding hydrophobic pocket formed of residues, Trp 741, Met 745, Leu 712, and Met 787 in both compounds, Figure 4.

### 2.4. O-Acetylated Analogues of Bicalutamide

**Scheme 4:**
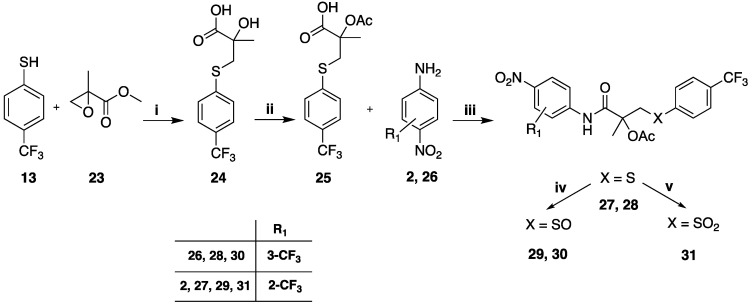
Reagents and conditions, (**i**) MeONa/MeOH, toluene, 60 °C, 3 h, then NaOH 10%; (**ii**) Ac_2_O, toluene, rt, 24 h; (**iii**) SOCl_2_, DMAP, toluene; (**iv**) *m*CPBA (1 equiv), DCM, 0 °C, 15–30 min; (**v**) *m*CPBA (2 equiv), DCM, rt, 24 h.

The acetyl derivatives (**27**, **28**) were prepared according to the route outlined in Scheme 4. The 4-trifluoromethyl thiophenol (**13**) was reacted with methyl 2-methylglycidate (**23**) followed by saponification using sodium hydroxide solution to form propionic acid derivative (**24**), which was then acetylated using acetic anhydride to afford (**25**). Coupling with aniline derivatives (**2**, **26**) was achieved using dimethylaminopyridine (DMAP) to afford **27** and **28** [21]. Sulfoxide derivatives (**29** and **30**) were prepared from **27** and **28**, respectively, using one equivalent of *m*CPBA at 0 °C for 15–30 min while the sulfone derivative (**31**) was prepared from **27** using two equivalents of *m*CPBA at room temperature for 24 h.

The antiproliferative activity of derivatives (**27**–**31**) showed that compound **27** displayed the most potent activity (IC_50_ = 4.69–15.03 µM) across the four human prostate cancer cell lines (22Rv1, DU-145, LNCaP, and VCap). Compounds **28** and **30** retained good activity, comparable (**28**) and better (**30**) than bicalutamide. However, compounds **29** and **31** did not possess antiproliferative activity (>100 µM), Table 4.

**Figure 5:**
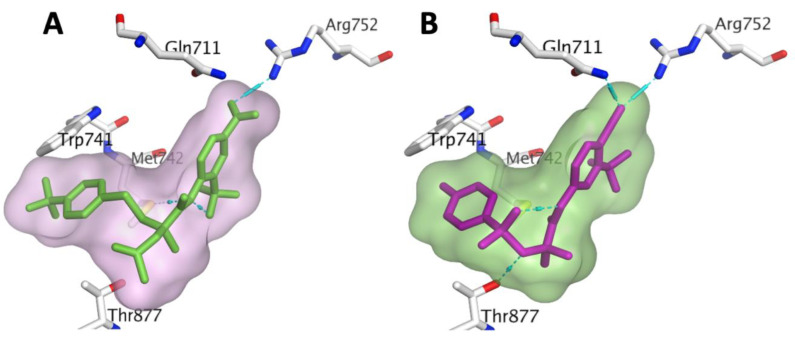
The predicted binding mode of compound **27** (**A**) and bicalutamide (**B**) inside the hAR-LBD showing H-bond interactions (turquoise dashed line) with key amino acids, Arg 752, Gln 711, Met 742, and Thr 877.

The predicted docking mode of compound **27** within the hAR-LBD showed H-bond interactions between the nitro group (NO_2_) and the guanidine group of Arg 752. In addition, hydrophobic interactions were observed between the 4-trifluoromethyl phenyl moiety and the surrounding hydrophobic pocket, π-π stacking between the terminal phenyl ring and the indole side chain of Trp 741. Also, the acetyl moiety of compound 27 seemed to occupy a small hydrophobic subpocket, Figure 5A. The binding mode of bicalutamide is shown in Figure 5B.

## 3. Materials and Methods

### 3.1. Chemistry

All chemicals were purchased from Sigma-Aldrich or Alfa Aesar and were used without further purification. Thin-layer chromatography (TLC): precoated aluminum-backed plates (60 F254, 0.2 mm thickness, Merck) were visualized under both short- and long-wave UV light (254 and 366 nm, respectively). Flash column chromatography was carried out using silica gel supplied by Fisher (60A, 35–70 mm); ^1^H-NMR (500 MHz), ^13^C-NMR (125 MHz), and ^19^F-NMR (470 MHz) spectra were recorded on a Bruker Avance 500 MHz spectrometer at 25 °C. Chemical shifts (δ) are expressed in parts per million (ppm) and coupling constants (J) are given in hertz (Hz). The following abbreviations are used in the assignment of NMR signals: s (singlet), bs (broad singlet), d (doublet), t (triplet), q (quartet), qn (quintet), m (multiplet), dd (doublet of doublet), dt (doublet of triplet), td (triple doublet), dq (double quartet), m (multiplet), dm (double multiplet). Reverse-phase HPLC, eluting with H_2_O/CH_3_CN from 90/10 to 0/100 in 30 min; flow = 1 mL/min, t_R_ = 20.39 min.

#### 3.1.1. General Method for the Preparation of Intermediates **4**–**5**

Methacryloyl chloride **3** (8.4 mL, 85.96 mmol) was added over the course of 10 min to a stirring solution of the appropriate trifluoromethylaniline **1**–**2** (10.75 mmol) in *N*,*N*-dimethylacetamide (10 mL) at room temperature for 24 h. After the reaction was complete, the mixture was diluted with ethyl acetate (100 mL) and extracted with saturated NaHCO_3_ solution (2 × 50 mL), then cold brine (2 × 50 mL). The combined organic layer was dried over anhydrous Na_2_SO_4_ and the solvent was removed under reduced pressure. The crude oil residue was purified by flash column chromatography eluting with chloroform-ethyl acetate 95:5 *v/v* to obtain the titled compounds.

*N-(4-cyano-3-(trifluoromethyl)phenyl)methacrylamide (**4**)* [22], Yield 92%, ^1^H-NMR (CDCl_3_) δ 8.10 (d, *J* = 2Hz, 1H, Ar*H*), 8.06 (bs, 1H, N*H*), 8.01 (dd, J = 2, 8.5 Hz, 1H, Ar*H*), 7.81 (d, *J* = 8.5Hz, 1H, Ar*H*), 5.89 (d, *J* = 1Hz, 1H, C*H*_2_), 5.62 (q, *J* = 1.5Hz, 1H, C*H*_2_), 2.10 (dd, *J* = 0.5, 1.5 Hz, 3H, C*H*_3_). ^19^F-NMR: (CDCl_3_) δ −62.23**.**

*N-(4-Nitro-2-(trifluoromethyl)phenyl)methacrylamide (**5**)* [22], Yield 94%, ^1^H-NMR (CDCl_3_) δ 8.73 (d, *J* = 9 Hz, 1H, Ar*H*), 8.46 (d, *J* = 3 Hz, 1H, Ar*H*), 8.37 (dd, *J* = 9 Hz, 2.5 Hz, 1H, Ar*H*), 8.17 (bs, 1H, N*H*), 5.85 (q, *J*= 0.5 Hz, 1H, C*H*_2_), 5.58 (q, *J* = 1.5 Hz, 1H, C*H*_2_), 2.15-2.13 (dd, J = 1, 1.5 Hz, 1H, C*H*_3_). ^19^F-NMR: (CDCl_3_) d-61.31.

#### 3.1.2. General Method for the Preparation of Intermediates **6**–**7**

To a stirred solution of the intermediate **4**–**5** (3 mmol) in DCM (7 mL) was added 30% hydrogen peroxide (3.6 mL, 32.03 mmol). The reaction mixture was put in a water bath at rt and trifluoroacetic anhydride (3.7 mL, 26.7 mmol) was added slowly to the mixture, which was then stirred for 24 h. The reaction mixture was transferred to a separating funnel using DCM (30 mL). The organic layer was washed with distilled water (20 mL), sat. aq. Na_2_S_2_O_3_ (4 × 20 mL), sat. aq. NaHCO_3_ (3 × 20 mL), and brine (20 mL), dried over Na_2_SO_4_, and concentrated at reduced pressure.

*N-(4-Cyano-3-(trifluoromethyl)phenyl)-2-methyloxirane-2-carboxamide (**6**)* [22].The data are in accordance with literature data. Obtained in 86% yield as a yellow solid. ^1^H-NMR (CDCl_3_): d 8.38 (bs, 1H), 8.00 (d, *J* = 2.1 Hz, 1H), 7.88 (dd, *J* = 8.5 Hz, 2.1 Hz, 1H), 7.78 (d, *J* = 8.5 Hz, 1H), 3.00 (s, 2H), 1.68 (s, 3H). 

*2-Methyl-N-(4-nitro-2-(trifluoromethyl)phenyl)oxirane-2-carboxamide (**7**).* Obtained in 71% yield as a yellow wax. ^1^H-NMR (CDCl_3_): d 8.92 (bs, 1H), 8.74 (d, *J* = 9.6 Hz, 1H), 8.53 (d, *J* = 2.5 Hz, 1H), 8.44 (dd, *J* = 9.6 Hz, 2.5 Hz, 1H), 3.04 (d, *J*= 4.6 Hz, 1H), 3.02 (d, *J* = 4.6 Hz, 1H), 1.72 (s, 3H). ^19^F-NMR (CDCl_3_): d −61.69 (s, 3F). ^13^C-NMR (CDCl_3_): d 169.2, 142.9, 140.4, 128.35 (m), 123.7, 122.3 (m), 121.6, 119.2 (m), 56.5, 53.9, 16.4.

#### 3.1.3. General Method for the Preparation of Compounds **10**–**12**, **22**–**29**

To a mixture of sodium hydride (NaH) (60% in mineral oil, 0.050 g, 1.23 mmol) in anhydrous THF (2 mL) at 0 °C under Ar atmosphere was added a solution of the differently substituted thiophenol **8**, **9**, **19**–**21** (1.11 mmol) in 1 mL of anhydrous THF. This mixture was stirred at rt for 20 min. A solution of the different intermediate 17–21 (0.74 mmol) in anhydrous THF (3 mL) was added slowly. The reaction mixture was stirred at rt for 24h.The mixture was then diluted with ethyl acetate (30 mL), washed with brine (15 mL) and water (30 mL), dried over Na_2_SO_4_, and concentrated under vacuum. The crude residue was purified by flash column chromatography.

*N-(4-cyano-3-(trifluoromethyl)phenyl)-2-hydroxy-2-methyl-3-(pyridin-2-ylthio) propanamide (**10**),* yield 79%, ^1^H-NMR (CDCl_3_) δ 9.64 (s, 1H, N*H*), 8.89 (s, 1H, Ar*H*), 8.39 (ddd, *J =* 1, 1.5, 5 Hz, 1H, Ar*H*), 8.13 (d, *J* = 2 Hz, 1H, Ar*H*), 8.00 (dd, *J* = 2, 8.5 Hz, 1H, Ar*H*), 7.91 (d, *J =* 8.5 Hz, 1H, Ar*H*), 7.61 (ddd, *J =* 2, 8, 8.5 Hz, 1H, Ar*H*), 7.37 (dt, *J =* 1, 8 Hz, 1H, Ar*H*), 7.17 (ddd, *J =* 1, 5, 7.5 Hz, 1H, Ar*H*), 3.61 (d, *J =* 15.5 Hz, 1H, C*H*_2_), 3.50 (d, *J* = 15 Hz, 1H, C*H*_2_), 1.63 (s, 3H, C*H*_3_); ^19^F-NMR (CDCl_3_) δ −62.16 (s, 3F); ^13^C-NMR (CDCl_3_) δ 175.18 (C=O), 158.85 (Ar*C*), 148.32 (Ar*C*H), 141.69 (Ar*C*), 137.45 (Ar*C*H), 135.80 (Ar*C*H), 133.92 (q, ^2^J_C-F_ = 32.5 Hz, Ar*C*), 123.55 (Ar*C*H), 122.19 (q, ^1^J_C-F_ = 271.3 Hz, *C*F*_3_*), 121.66 (Ar*C*H), 120.92 (Ar*C*H), 117.16 (q, ^3^J_C-F_ = 5 Hz, Ar*C*H), 115.67 (Ar*C*), 104.15 (CN), 77.01 (*C*OH), 41.48 (*C*H_2_), 26.81 (*C*H_3_). MS [ESI, *m/z*]: 382.1 [M+H]^+^, 404.1 [M+Na]^+^. Reverse-phase HPLC, eluting with H_2_O/CH_3_CN from 90/10 to 0/100 in 30 min; flow = 1 mL/min, t_R_ = 21.17 min 99.5%.

*2-Hydroxy-2-methyl-N-(4-nitro-2-(trifluoromethyl)phenyl)-3-(pyridin-2-ylthio) propenamide (**11**),* yield 75%, ^1^H-NMR (CDCl_3_) δ 10.26 (s, 1H, N*H*), 9.08 (s, 1H, Ar*H*), 8.84 (d, *J* = 9 Hz, 1H, Ar*H*), 8.52 (d, *J* = 3 Hz, 1H, Ar*H*), 8.42 (m, 2H, Ar*H*), 7.61 (m, 1H, Ar*H*), 7.37 (d, *J* = 8.5 Hz, 1H, Ar*H*), 7.18 (m, 1H, Ar*H*), 3.60 (d, *J* = 15 Hz, 1H, C*H*_2_), 3.52 (d, *J* = 15 Hz, 1H, C*H*_2_), 1.65 (s, 3H, C*H*_3_); ^19^F-NMR (CDCl_3_) δ −62.01 (s, 3F); ^13^C-NMR (CDCl_3_) δ 175.24 (C=O), 158.68 (Ar*C*), 148.31 (Ar*C*H), 142.62 (Ar*C*), 141.00 (Ar*C*), 137.40 (Ar*C*H), 128.27 (Ar*C*H), 123.36 (Ar*C*H), 122.78 (q, ^1^J_C-F_ = 272 Hz, *C*F*_3_*), 122.36 (q, ^3^J_C-F_ = 5.5 Hz, Ar*C*H), 122.04 (Ar*C*H), 120.86 (Ar*C*H), 119.28 (q, ^2^J_C-F_ = 32.5 Hz, Ar*C*), 77.13 (*C*OH), 41.46 (*C*H_2_), 26.54 (*C*H_3_). MS (ES+) *m/z*: 402.1 [M + H]^+^ 424.1 [M + Na]^+^. Reverse-phase HPLC, eluting with H_2_O/CH_3_CN from 90/10 to 0/100 in 30 min; flow = 1 mL/min, t_R_ = 23.98 min 99.75%.

*2-Hydroxy-2-methyl-N-(4-nitro-2-(trifluoromethyl)phenyl)-3-(4-(trifluoromethyl) pyrimidin-2-ylthio)propanamide (**12**),* yield 73%, ^1^H-NMR (CDCl_3_) δ 10.02 (s, 1H, NH), 8.83 (d, *J* = 5 Hz, 1H, ArH), 8.80 (d, *J* = 9.5 Hz, 1H, ArH), 8.53 (d, *J* = 2.5 Hz, 1H, ArH), 8.45 (dd, *J* = 2.5, 9 Hz, 1H, ArH), 7.46 (d, *J* = 5 Hz, 1H, ArH), 6.43 (s, 1H, OH), 3.69 (s, 2H, CH_2_), 1.68 (s, 3H, CH_3_); ^19^F-NMR (CDCl_3_) δ −62.00, -70.26; ^13^C-NMR (CDCl_3_) δ 174.21 (ArC), 173.91 (C=O), 159.79 (ArCH), 156.02 (q, ^2^J_C-F_ = 36.3 Hz, ArC), 142.87 (ArC), 140.67 (ArC), 128.31 (ArCH), 122.76 (q, ^1^J_C-F_ = 271.9 Hz, CF_3_), 122.39 (q, ^3^J_C-F_ = 5.5 Hz, ArCH), 122.30 (ArCH), 119.79 (q, ^1^J_C-F_ = 274 Hz, CF_3_), 119.49 (q, ^2^J_C-F_ = 31.6 Hz, ArC), 112.97 (q, ^3^J_C-F_ = 2.6 Hz, ArCH), 77.82 (COH), 40.67 (CH_2_), 26.38 (CH_3_).MS (ES+) m/z: 471.1 [M + H]^+^, 493.1 [M + Na]^+^. t_R_ = 23.16 min 95.22%. 

#### 3.1.4. General Method for the Preparation of Compounds **16**–**18**

To a mixture of 60% sodium hydride (NaH) in mineral oil (2.36 mmol) in 5 mL of anhydrous tetrahydrofuran at 0 °C under anhydrous THF under nitrogen atmosphere was added dropwise the corresponding thiophenol **10**–**13** (2.05 mmol). This mixture was stirred at room temperature for 20 min. A solution of the intermediate **5** (1.57 mmol in 5 mL anhydrous tetrahydrofuran) was added slowly to the thiophenol mixture and stirred at room temperature for 24h. The mixture was concentrated under vacuum, then diluted with 30 mL ethyl acetate washed with 20 mL brine and 30 mL water, dried over anhydrous sodium sulfate, and concentrated under vacuum. The crude residue was purified by column chromatography eluting with chloroform-ethyl acetate gradually increasing from 95:5 to 90:10 *v/v* [12].

*2-Methyl-N-(4-nitro-2-(trifluoromethyl) phenyl)-3-(4-(trifluoromethyl)phenylthio) propanamide (**16**),* yield 71%, ^1^H-NMR (CDCl_3_) δ 8.64 (d, *J* = 9 Hz, 1H, Ar*H*), 8.54 (d, *J* = 3 Hz, 1H, Ar*H*), 8.42 (dd, *J* = 3, 9.5 Hz, 1H, Ar*H*), 7.80 (s, 1H, O*H*), 7.55 (d, *J* = 8 Hz, 2H, Ar*H*), 7.44 (d, *J* = 8 Hz, 2H, Ar*H*), 3.43 (dd, *J =* 8.5, 14 Hz, 1H, C*H*_2_), 3.16 (dd, *J =* 5.5, 13.5 Hz, 1H, C*H*_2_), 2.73 (m, 1H, C*H*), 1.44 (d, *J =* 7 Hz, 3H, C*H*_3_); ^19^F-NMR (CDCl_3_) δ: −62.63, −61.00; ^13^C-NMR (CDCl_3_) δ*:* 172.73 (*C*=O), 143.04 (Ar*C*), 141.71 (Ar*C*), 140.56 (Ar*C*), 128.49 (Ar*C*H), 128.26 (Ar*C*H), 128.02 (m, Ar*C*), 125.93 (q, ^3^J_C-F_ = 3.7 Hz, Ar*C*H), 125.87, 123.30 (Ar*C*H), 123.73 (m, 2*C*F*_3_*), 122.31 (q, ^3^J_C-F_ = 5 Hz, Ar*C*H), 120.65 (m, Ar*C*), 43.09 (*C*H), 36.32 (*C*H_2_), 17.46 (*C*H_3_). MS (ES+) *m/z*: 453.1 [M + H]^+^, 475.1 [M + Na]^+^. Reverse-phase HPLC, eluting with H_2_O/CH_3_CN from 90/10 to 0/100 in 30 min; flow = 1 mL/min, t_R_ = 24.45 min 98.0%.

*2-Methyl-N-(4-nitro-2-(trifluoromethyl)phenyl)-3-(2-(trifluoromethoxy)phenylthio) propanamide (**17**),* yield 69%, ^1^H-NMR (CDCl_3_) δ 8.65 (d, *J* = 9.5 Hz, 1H, Ar*H*), 8.54 (d, *J* = 2.5 Hz, 1H, Ar*H*), 8.43 (dd, *J* = 3, 9.5 Hz, 1H, Ar*H*), 7.80 (s, 1H, N*H*), 7.48 (m, 1H, Ar*H*), 7.23 (m, 3H, Ar*H*), 3.37 (dd, *J =* 8, 13.5 Hz, 1H, C*H_2_*), 3.08 (dd, *J =* 5.5, 13.5 Hz, 1H, C*H_2_*), 2.68 (m, 1H, C*H*), 1.42 (d, *J =* 7 Hz 3H, C*H_3_*); ^19^F-NMR (CDCl_3_) δ −61.13, −57.29; ^13^C-NMR (CDCl_3_) δ 172.90 (*C*=O), 160.91 (Ar*C*), 143.04 (Ar*C*), 140.30 (Ar*C*), 133.91 (Ar*C*H), 131.48 (Ar*C*H), 129.45 (m, *C*F*_3_*), 128.22 (Ar*C*H), 127.36 (Ar*C*H), 123.96 (m, *C*F*_3_*), 123.48 (Ar*C*H), 122.30 (q, ^3^J_C-F_ = 5 Hz, Ar*C*H), 121.55 (m, Ar*C*), 121.25 (Ar*C*H), 120.58 (Ar*C*), 43.12 (*C*H), 36.58 (*C*H_2_), 17.37 (*C*H_3_).MS (ES+) *m/z*: 469.1 [M + H]^+^, 491.1 [M + Na]^+^. t_R_ = 24.22 min 95.02%.

*3-(2,4-Difluorophenylthio)-2-methyl-N-(4-nitro-2-(trifluoromethyl)phenyl) propanamide (**18**),* yield 75%, ^1^H-NMR (CDCl_3_) δ 8.68 (d, *J* = 9.5 Hz, 1H, Ar*H*), 8.55 (d, *J* = 2.5 Hz, 1H, Ar*H*), 8.44 (dd, *J* = 2.5, 9 Hz, 1H, Ar*H*), 7.85 (s, 1H, N*H*), 7.47 (m, 1H, Ar*H*), 6.88 (m, 2H, Ar*H*), 3.27 (dd, *J =* 8.5, 14 Hz, 1H, *C*H_2_), 2.99 (dd, *J =* 5.5, 14 Hz, 1H, *C*H_2_), 2.64 (m, 1H, *C*H), 1.38 (d, *J =* 7 Hz, 3H, *C*H_3_); ^19^F-NMR (CDCl_3_) δ −61.06 (3F), −102.93 (1F), −108.49 (1F); ^13^C-NMR (CDCl_3_) δ 172.98 (*C*=O), 164.15 (Ar*C*), 158.55 (Ar*C*), 142.99 (Ar*C*), 140.77 (Ar*C*), 135.81 (m, Ar*C*H), 135.73, 128.25 (Ar*C*H), 124.89 (q, ^1^J_C-F_ = 228 Hz, Ar*C*), 123.99 (ArC), 123.29 (Ar*C*H), 122.29 (q, ^3^J_C-F_ = 6.3 Hz, Ar*C*H), 121.87 (m, ArC), 112.14 (dd, ^4^J_C-F_ = 3.6 Hz, ^2^J_C-F_ = 22.5 Hz, Ar*C*H), 104.82 (t, ^2^J_C-F_ = 25.9 Hz, Ar*C*H), 43.54 (*C*H), 38.28 (*C*H_2_), 17.38 (*C*H_3_). MS (ES^+^) *m/z*: 421.1 [M + H]^+^, 443.1 [M + Na]^+^. Reverse-phase HPLC, eluting with H_2_O/CH_3_CN from 90/10 to 0/100 in 30 min; flow = 1 mL/min, t_R_ = 24.92 min 95.10%.

#### 3.1.5. General Method for the Preparation of Sulfone Compounds **19**–**22**, **31**

To a stirring solution of the different sulfide **10**, **11**, **16, 17, 27** (0.7 mmol) in 5 mL anhydrous dichloromethane (DCM) was added *m*-chloroperbenzoic acid (*m*CPBA) (1.4 mmol). The solution was stirred at room temperature for 24 h. The reaction mixture was neutralized with 1M sodium hydroxide. Distilled water (50 mL) was added to the reaction mixture and was extracted with 2 × 50 mL of dichloromethane. The combined organic layers were washed, dried over anhydrous sodium sulfate, and concentrated in vacuo. The crude residue was purified by column chromatography, preparative TLC, or crystallization from methanol. 

*N-(4-cyano-3-(trifluoromethyl)phenyl)-2-hydroxy-2-methyl-3-(pyridin-2-ylsulfonyl)propanamide (**19**),* yield 76%, ^1^H-NMR (CDCl_3_) δ 9.32 (s, 1H, N*H*), 8.74 (d, *J =* 5 Hz, 1H, Ar*H*), 8.13 (d, *J =* 8 Hz, 1H, Ar*H*), 8.07 (m, 2H, Ar*H*), 7.96 (d, *J* = 7.5 Hz, 1H, Ar*H*), 7.81 (d, *J =* 8 Hz, 1H, Ar*H*), 7.68 (m, 1H, Ar*H*), 6.86 (s, 1H, O*H*), 4.38 (d, *J =* 15.5 Hz, 1H, C*H_2_*), 3.76 (d, *J* = 15 Hz, 1H, C*H_2_*), 1.64 (s, 3H, C*H_3_*); ^19^F-NMR (CDCl_3_) δ −62.17; ^13^C-NMR (CDCl_3_) δ*:* 172.72 (*C*=O), 155.12 (Ar*C*), 149.46 (Ar*C*H), 141.50 (Ar*C*), 139.43 (Ar*C*H), 135.82 (Ar*C*H), 136.55 (m, *C*F_3_), 128.21 (Ar*C*H), 121.98 (Ar*C*H), 121.93 (Ar*C*H), 121.42 (Ar*C*), 117.37 (q, ^3^J_C-F_ = 5 Hz, Ar*C*H), 115.54 (*C*N), 99.99 (Ar*C*), 73.45 (*C*OH), 60.99 (*C*H_2_), 27.77 (*C*H_3_). MS (ES+) *m/z*: 414.1 [M + H]^+^, 436.1 [M + Na]^+^. Reverse-phase HPLC, eluting with H_2_O/CH_3_CN from 90/10 to 0/100 in 30 min; flow = 1 mL/min, t_R_ = 15.42 min 98.7%. 

*2-Hydroxy-2-methyl-N-(4-nitro-2-(trifluoromethyl)phenyl)-3-(pyridin-2-yl sulfonyl) propanamide, (**20**),* yield 81%, ^1^H-NMR (CDCl_3_) δ 9.88 (s, 1H, N*H*), 8.77 (d, *J* = 5 Hz, 1H, Ar*H*), 8.71 (d, *J* = 9 Hz, 1H, Ar*H*), 8.55 (d, *J* = 2.5 Hz, 1H, Ar*H*), 8.44 (dd, *J* = 3, 9.5 Hz, 1H, Ar*H*), 8.14 (d, *J* = 7.5 Hz, 1H, Ar*H*), 7.70 (m, 1H, Ar*H*), 8.09 (m, 1H, Ar*H*), 7.09 (s, 1H, O*H*), 4.39 (d, *J* = 15.5 Hz, 1H, C*H_2_*), 3.81 (d, *J* = 15.5 Hz, 1H, C*H_2_*), 1.65 (s, 3H, C*H_3_*); ^19^F-NMR (CDCl_3_) δ −61.68; ^13^C-NMR (CDCl_3_) δ 172.90 (*C*=O), 157.37 (Ar*C*), 149.35 (Ar*C*H), 142.95 (Ar*C*), 140.82 (Ar*C*), 139.55 (Ar*C*H), 128.25 (Ar*C*H), 123.91 (m, Ar*C*), 123.01 (Ar*C*H), 122.72 (Ar*C*H), 122.38 (m, *C*F_3_) 122.34 (q, ^3^J_C-F_ = 5.6 Hz, Ar*C*H), 121.79 (Ar*C*H), 73.37 (*C*OH), 61.37 (*C*H_2_), 27.64 (*C*H_3_).MS (ES+) *m/z*: 434.1 [M + H]^+^, 456.1 [M + Na]^+^. t_R_ = 17.44 min 99.47%. 

*2-Methyl-N-(4-nitro-2-(trifluoromethyl)phenyl)-3-((4-(trifluoromethyl)phenyl) sulfonyl) propenamide (**21**),* yield 74.7%, ^1^H-NMR (CDCl_3_) δ 8.57 (d, *J* = 2 Hz, 1H, Ar*H*), 8.46 (d, *J* = 9 Hz, 1H, Ar*H*), 8.43 (dd, *J* = 9.5, 2.5 Hz, 1H, Ar*H*), 8.10 (d, *J* = 8 Hz, 2H, Ar*H*), 7.86 (m, 3H, Ar*H,* N*H*), 3.78 (dd, *J =* 4, 9.5 Hz, 1H, C*H*_2_), 3.70 (dd, *J =* 4.5, 10.5 Hz, 1H, C*H*_2_), 3.12 (m, 1H, C*H*), 1.39 (d, *J =* 7.5 Hz, 3H, C*H*_3_); ^19^F-NMR (CDCl_3_) δ −60.92, −63.33; ^13^C-NMR (CDCl_3_) δ 171.48 (*C*=O), 150.72 (Ar*C*), 143.42 (Ar*C*), 140.31 (Ar*C*), 135.77, 128.69 (Ar*C*H), 128.21 (Ar*C*H), 126.64 (q, ^3^J_C-F_ = 3.6 Hz, Ar*C*H), 124.06 (Ar*C*H), 124.74, 124.70, 124.67, 124.47, 124.42, 124.39, 122.40, 122.36 (q, ^3^J_C-F_ = 5.4 Hz, Ar*C*H), 120.8 (q, ^1^J_C-F_ = 232.8 Hz, *C*F_3_), 120.32, 58.70, 37.14 (*C*H_2_), 18.57 (*C*H_3_). MS (ES+) *m/z*: 485.1 [M + H]^+^, 507.1 [M + Na]^+^. Reverse-phase HPLC, eluting with H_2_O/CH_3_CN from 90/10 to 0/100 in 30 min; flow = 1 mL/min, t_R_ = 21.79 min 97.30%.

*2-Methyl-N-(4-nitro-2-(trifluoromethyl)phenyl)-3-(2-(trifluoromethoxy) phenylsulfonyl) propanamide (**22**),* yield 71.6%, ^1^H-NMR (CDCl_3_) δ 8.55 (d, *J* = 2.5 Hz, 1H, Ar*H*), 8.49 (d, *J* = 9 Hz, 1H, Ar*H*), 8.42 (dd, *J* = 2.5, 9 Hz, 1H, Ar*H*), 8.08 (dd, *J* = 2, 8 Hz, 1H, Ar*H*), 7.88 (s, 1H, N*H*), 7.73 (ddd, *J* = 2, 7.5, 8.5 Hz, 1H, Ar*H*), 7.49 (2m, 1H, Ar*H*), 7.45 (td, *J =* 1, 7.5 Hz, 1H, Ar*H*), 4.01 (dd, *J =* 8.5, 14 Hz, 1H, C*H*_2_), 3.39 (dd, *J =* 4, 14 Hz, 1H, C*H*_2_), 3.12 (m, 1H, C*H*), 1.48 (d, *J =* 7.5 Hz, 3H, C*H*_3_); ^19^F-NMR (CDCl_3_) δ −60.99, −56.24; ^13^C-NMR (CDCl_3_) δ 171.28 (*C*=O), 160.35 (Ar*C*), 143.61 (Ar*C*), 140.61 (Ar*C*), 135.96 (Ar*C*H), 130.98 (Ar*C*H), 129.50 (Ar*C*), 129.16 (Ar*C*), 128.13 (Ar*C*H), 126.79 (Ar*C*H), 123.90 (Ar*C*H), 123.27 (*C*F*_3_*), 121.45 (Ar*C*), 120.26 (Ar*C*H), 120.24 (Ar*C*H), 58.28 (*C*H_2_), 37.21 (*C*H), 18.37 (*C*H_3_). MS (ES+) *m/z*: 501.1 [M + H]^+^, 523.0 [M + Na]^+^. Reverse-phase HPLC, eluting with H_2_O/CH_3_CN from 90/10 to 0/100 in 30 min; flow = 1 mL/min, t_R_ = 24.22 min 95.00%.

#### 3.1.6. General Method for the Preparation of Acetylated Compounds **27**, **28**

4-trifluoromethyl thiophenol (**13**, 0.283 g, 0.0178 moles) was placed in a round-bottomed flask and heated to 55–60 °C.; methyl 2-methylglycidate (**23**, 2.11 g, 0.0181 moles) was then added slowly over an hour at 55–60 °C after a further 60 min at 55–60 °C. TLC showed that the reaction was complete with major presence of the methyl ester intermediate. Water was added, followed by 30% NaOH solution (2.61 g, 0.0195 moles) and the mixture was heated at 60 °C for 2 h. TLC showed only the spot of the desired 2-hydroxy 2-methyl-3-(4-trifluoromethylphenylthio) propionic acid (**24**). To a solution of 24 (1.36 g, 0.0593 moles) in 7 mL of anhydrous toluene was added acetic anhydride (0.601 mL, 0.00623 moles) and the resulting solution was heated at 100 °C for 5 h. After cooling to room temperature, the solution was washed twice with 2 mL of water, and the combined organic phases were dried and removed under reduced pressure. A dense oil was obtained, which solidified spontaneously (**25**). To a solution of the acetoxy derivative (**25**) (0.4 g, 0.00146 moles) in 20 mL of anhydrous toluene at rt was added 0.127 mL (0.00148 moles) of thionyl chloride, and the mixture was heated at 85–90 °C for 3–4 h. The solvent was completely evaporated under reduced pressure. The product was obtained as an oil which was used as is in the successive reaction. To a solution of the acid chloride (0.21g, 0.00072 moles) in 1.2 mL of anhydrous toluene was added 0.09 g (0.00072 moles) of DMAP, maintaining the solution at room temperature; the suspension was allowed to react for 10 min, then 0.115 g (0.00062 moles) of the corresponding 4-amino-benzonitrile derivative (**2**, **26)** dissolved in toluene was added and the mixture was heated at 75–80 °C for 8–10 h following the disappearance of the amine by TLC (eluent: ethyl acetate/toluene 75/25). After cooling to room temperature, the solution was diluted with toluene and treated with a 5% solution of HCl, separated, and washed with 5% bicarbonate solution. The organic phase was cured and evaporated under reduced pressure. Crude propionamide derivatives (**48** and **49**) were obtained in quantitative yield [21].

*2-Methyl-l-(4-nitro-2-(trifluoromethyl) phenylamino)-l-oxo-3-(4-(trifluoromethyl) phenylthio)propan-2-yl acetate (**27**),* yield 30%, ^1^H-NMR (CDCl_3_) δ 9.10 (s, 1H, N*H*), 8.62 (d, *J* = 9 Hz, 1H, Ar*H*), 8.54 (d, *J* = 2.5 Hz, 1H, Ar*H*), 8.40 (dd, *J =* 3, 9 Hz, 1H, Ar*H*), 7.45 (m, 4H, Ar*H*), 4.07 (d, *J =* 14.5 Hz, 1H, C*H_2_*), 3.75 (d, *J* = 14 Hz, 1H, C*H_2_*), 1.99 (s, 3H, C*H_3_*), 1.89 (s, 3H, C*H_3_*); ^19^F-NMR (CDCl_3_) δ −61.29, −62.78; ^13^ C-NMR (CDCl_3_) δ 169.90 (C=O), 168.21 (C=O), 143.05 (Ar*C*), 140.03 (Ar*C*), 139.45 (Ar*C*), 130.22 (Ar*C*H), 128.90 (q, ^2^J_C-F_ = 32.6 Hz, Ar*C*), 128.49 (Ar*C*H), 125.65 (q, ^3^J_C-F_ = 3.8 Hz, Ar*C*H), 123.79 (q, ^1^J_C-F_ = 270.6 Hz, *C*F*_3_*), 123.03 (q, ^1^J_C-F_ = 272.1 Hz, *C*F*_3_*), 122.34 (q, ^3^J_C-F_ = 6.3 Hz, Ar*C*H) 121.93, 121.89 (Ar*C*H), 118.82 (q, ^2^J_C-F_ = 31.3 Hz, Ar*C*), 84.34 (*C*OAc), 39.06 (*C*H_2_), 22.94 (*C*H_3_), 21.14 (*C*H_3_). MS (ES+) *m/z*: 511.1 [M + H]^+^, 533.1 [M + Na]^+^. Reverse-phase HPLC, eluting with H_2_O/CH_3_CN from 90/10 to 0/100 in 30 min; flow = 1 mL/min, t_R_ = 25.50 min 97.3%.

*2-Methyl-l-(4-nitro-3-(trifluoromethyl) phenylamino)-l-oxo-3-(4-(trifluoromethyl) phenylthio) propan-2-yl acetate (**28**),* yield 84%, ^1^H-NMR (CDCl_3_) δ 1.90 (s, 3H), 2.10 (s, 3H), 3.77 (d, *J* = 14 Hz, 1H), 4.04 (d, *J =* 14 Hz, 1H), 7.44 (d, *J =* 8.5 Hz, 2H), 7.48 (d, *J =* 8.5 Hz, 2H), 7.88 (d, *J =* 2 Hz, 1H), 7.92 (dd, *J* = 2, 8.5 Hz, 1H), 7.97 (d, *J* = 9 Hz, 1H), 8.40 (s, 1H); ^19^F-NMR (CDCl_3_) δ −62.80, −60.14; ^13^C-NMR (CDCl_3_) δ 169.84 (C=O), 168.59 (C=O), 144.75 (Ar*C*), 139.59 (Ar*C*), 129.53 (Ar*C*H), 129.11 (q, ^2^J_C-F_ = 31.5 Hz, Ar*C*), 126.95 (Ar*C*H), 125.75 (q, ^3^J_C-F_ = 3.8 Hz, Ar*C*H), 124.84 (q, ^1^J_C-F_ = 270.6 Hz, *C*F*_3_*), 123.43 (q, ^1^J_C-F_ = 271.2 Hz, *C*F*_3_*), 122.56 (Ar*C*H) 118.51 (Ar*C*H), 118.50 (q, ^2^J_C-F_ = 30.4 Hz, Ar*C*), 83.89 (*C*OAc), 60.39 (*C*H_2_), 22.95 (*C*H_3_), 21.69 (*C*H_3_). MS (ES+) *m/z*: 511.1 [M + H]^+^, 533.1 [M + Na]^+^. Reverse-phase HPLC, eluting with H_2_O/CH_3_CN from 90/10 to 0/100 in 30 min; flow = 1 mL/min, t_R_ = 24.40 min 96.31%.

*2-Methyl-l-(4-nitro-2-(trifluoromethyl) phenylamino)- l-oxo-3-(4-(trifluoromethyl) phenylsulfinyl)propan-2-yl acetate (**29**),* yield 69%, ^1^H-NMR (CDCl_3_) δ [9.10 (s), 9.26 (s), 1H, N*H*], [8.73 (s), 8.75 (s), 1H, Ar*H*], [8.57 (d, *J* = 2.5 Hz), 8.61 (d, *J* = 2.5 Hz), 1H, Ar*H*], [8.47 (dd, *J* = 2.5, 9.5 Hz), 8.51 (dd, *J* = 2.5, 9.5 Hz), 1H, Ar*H*), 7.81 (m, 4H, Ar*H*), [3.62 (d, *J* = 14 Hz), 3.82 (s), 3.98 (d, *J* = 14 Hz), 2H, C*H_2_*], [2.25 (s), 2.36 (s), 3H, C*H_3_*], [1.93 (s), 1.96 (s), 3H, C*H_3_*]; ^19^F-NMR (CDCl_3_) δ −61.29, −62.92; ^13^ C-NMR (CDCl_3_) δ 168.98 (C=O), 168.88 (C=O), 147.83 (Ar*C*), (143.32, 142.47, Ar*C*), (140.30, 139.97, Ar*C*), 133.44 (m, Ar*C*), 128.52 (Ar*C*H), 126.47 (m, Ar*C*H), 125.84 (q, ^1^J_C-F_ = 263.4 Hz, *C*F_3_), [124.49, 124.43, Ar*C*H], [123.13, 123.00, Ar*C*H], [122.54 (q, ^3^J_C-F_ = 5 Hz), 122.40 (q, ^3^J_C-F_ = 6.3 Hz), Ar*C*H], 121.02 (q, ^1^J_C-F_ = 226.9 Hz, *C*F_3_), (82.06, 81.37, *C*OCH_3_), (62.65, 61.75, *C*H_2_), (24.34, 23.98, *C*H_3_), (21.64, 21.61, *C*H_3_). MS (ES+) *m/z*: 527.1 [M + H]^+^, 549.1 [M + Na]^+^. Reverse-phase HPLC, eluting with H_2_O/CH_3_CN from 90/10 to 0/100 in 30 min; flow = 1 mL/min, t_R_ = 22.54 min 98.81%.

*2-Hydroxy-2-methyl-N-(4-nitro-3-(trifluoromethyl)phenyl)-3-(4-(trifluoromethyl) phenylsulfinyl) propanamide (**30**),* yield 59%, ^1^H-NMR (CDCl_3_) δ 9.47 (s, 1H, N*H*), 8.26 (d, *J* = 1.5 Hz, 1H, Ar*H*), 8.06 (m, 2H, Ar*H*), 7.89 (d, *J* = 8.5 Hz, 2H, Ar*H*), 7.83 (d, *J* = 8 Hz, 2H, Ar*H*), 5.89 (s, 1H, O*H*), 3.61 (d, *J =* 13 Hz, 1H, C*H_2_*), 3.08 (d, *J* = 13 Hz, 1H, C*H_2_*), 1.62 (s, 3H, C*H_3_*); ^19^F-NMR (CDCl_3_) δ −60.10, −63.02; ^13^C-NMR (CDCl_3_) δ 173.26 (C=O), 149.51 (Ar*C*), 145.87 (Ar*C*), 141.21 (Ar*C*), 137.77 (m, Ar*C*), 126.84 (q, ^3^J_C-F_ = 3.4 Hz, Ar*C*H), 124.64 (Ar*C*H), 124.35 (Ar*C*), 124.24 (Ar*C*H), 122.59 (Ar*C*), 122.32 (Ar*C*H), 120.64 (m, Ar*C*), 118.50 (q, ^3^J_C-F_ = 5 Hz, Ar*C*H), 76.80 (*C*OH), 62.25 (*C*H_2_), 28.25 (*C*H_3_). MS (ES+) *m/z*: 485.1 [M + H]^+^, 507.1 [M + Na]^+^. Reverse-phase HPLC, eluting with H_2_O/CH_3_CN from 90/10 to 0/100 in 30 min; flow = 1 mL/min, t_R_ = 18.95 min 96.76%.

*2-Methyl-l-(4-nitro-2-(trifluoromethyl) phenylamino)-l-oxo-3-(4-(trifluoromethyl) phenylsulfonyl) propan-2-yl acetate (**31**),* yield 83%, ^1^H-NMR (CDCl_3_) δ 9.22 (s, 1H, N*H*), 8.71 (d, *J* = 9.5 Hz, 1H, Ar*H*), 8.60 (d, *J* = 2.5 Hz, 1H, Ar*H*), 8.49 (dd, *J* = 2.5, 9 Hz, 1H, Ar*H*), 8.06 (d, *J =* 8 Hz, 2H, Ar*H*), 7.84 (d, *J =* 8.5 Hz, 2H, Ar*H*), 4.41 (d, *J =* 14.5 Hz, 1H, C*H_2_*), 4.07 (d, *J* = 14.5 Hz, 1H, C*H_2_*), 2.26 (s, 3H, C*H_3_*), 1.90 (s, 3H, C*H_3_*); ^19^F-NMR (CDCl_3_) δ −63.31, −61.29; ^13^ C-NMR (CDCl_3_) δ 169.05 (*C*=O), 168.72 (*C*=O), 143.39 (Ar*C*), 143.02 (Ar*C*), 140.14 (Ar*C*), 136.06 (m, Ar*C*), 128.75 (Ar*C*H), 128.54 (Ar*C*H), 126.57 (q, ^3^J_C-F_ = 3.8 Hz, Ar*C*H), 124.07 (m, 2*C*F_3_), 122.82 (Ar*C*H), 122.47 (q, ^3^J_C-F_ = 5 Hz, Ar*C*H), 121.09 (m, Ar*C*), 80.16 (*C*OAc), 57.74 (*C*H_2_), 24.36 (*C*H_3_), 21.72 (*C*H_3_). MS (ES+) *m/z*: 543.1 [M + H]^+^, 565.1 [M + Na]^+^ Reverse-phase HPLC, eluting with H_2_O/CH_3_CN from 90/10 to 0/100 in 30 min; flow = 1 mL/min, t_R_ = 24.08 min 96.52%.

### 3.2. Cell Growth Inhibition Viability Assay

All bicalutamide derivatives were screened for their antiproliferative activity using the Oncotest monolayer assay against four human prostate cancer cell lines (22Rv1, DU-145, LNCaP, and VCap). Bicalutamide and enzalutamide were used as positive controls. 

A modified propidium iodide (PI)-based monolayer assay was used to assess the anticancer activity of the compounds. Briefly, cells were harvested from exponential phase cultures, counted, and plated in 96-well flat-bottom microtiter plates at a cell density of 8000–12,000 cells/well. After a 24 h recovery period to allow the cells to resume exponential growth, 10 mL of culture medium (six control wells/plate) or culture medium with test compound was added. The compounds were applied in half-log increments at 10 concentrations in triplicate. After a total treatment period of 96 h, cells were washed with 200 mL PBS to remove dead cells and debris. Then, 200 mL of a solution containing 7 mg/mL propidium iodide (PI) and 0.1% (*v/v*) Triton X-100 was added. After an incubation period of 1–2 h at room temperature, fluorescence (FU) was measured using the EnSpire Multimode Plate Reader (excitation l = 530 nm, emission l = 620 nm) to quantify the amount of attached viable cells. IC_50_ values were calculated by four-parameter nonlinear curve fit using Oncotest Warehouse Software. For calculation of mean IC_50_ values, the geometric mean was used [23]. 

### 3.3. Docking Studies

The X-ray crystal structure of the human androgen receptor ligand-binding domain hAR-LBD was downloaded from the Protein Data Bank (PDB code; 3RLJ) [24] and prepared for docking using the MOE (Molecular Operating Environment) [25] protein preparation tools. The chemical structures of our compounds were constructed, rendered, and minimized with the MMFF94x force-field in MOE. The docking simulations were performed using the Glide SP within Maestro software using the default settings (Glide, version 9.5, Schrodinger) [26]. The docking output database was saved as a mol2 file, and the visual inspection of the docking modes was performed in MOE.

## 4. Conclusions

Fifteen bicalutamide derivatives were prepared and their antiproliferative activity was evaluated in vitro against four different human prostate cancer cell lines (22Rv1, DU-145, LNCaP, and VCap). These modifications offer an insight into the SAR of various propionanilide analogues. Bicalutamide and enzalutamide were used as positive controls. The results summarized in Table 1, Table 2, Table 3 and Table 4 indicated that seven compounds, sulfide (**12**), deshydroxy (**16**, **17**), sulfone (**21**), and acetylated (**27**) derivatives, have better antiproliferative activity than the positive controls, bicalutamide (IC_50_ = 45.20–51.61 µM*)* and enzalutamide (IC_50_ = 11.47–53.04 µM). The deshydroxy analogue (**16**) was the most active compound with IC_50_ = 6.59–10.86 µM, followed by the acetylated derivative (**27**) with IC_50_ = 4.69–15.03 µM across the four prostate cancer cell lines. Molecular modeling was used to find a plausible explanation of the drop in activity upon oxidation of the sulfide analogue (**16**) into sulfone counterpart (**21**). The dose response curves of compounds (**16, 17**) and their oxidized analogues (**21**, **22**) are represented in Figure 3. Retention and enhancement of bicalutamide antiproliferative activity were observed in some compounds. Displaying antiproliferative activity in the 22Rv1 and DU-145 cell lines suggests that other antiproliferative mechanisms could be involved. This possible off-target effect was noticed as well in the parent bicalutamide, which showed similar IC_50_ values across the four cell lines. These findings may serve as a useful starting point for developing novel AR modulators.

## Figures and Tables

**Table 1:** In vitro antiproliferative activity of the sulfide analogues of bicalutamide (**10**–**12**) across four human prostate cancer cell lines (DU-145, 22Rv, LNCaP, and VCap). All data are mean values from experiments carried out on three separate occasions.

ID	22Rv1 IC_50_ (µM)	DU-145 IC_50_ (µM)	LNCaP IC_50_ (µM)	VCap IC_50_ (µM)
**10**	27.94	40.47	23.51	51.61
**11**	>100	>100	16.34	>100
**12**	17.42	43.54	16.88	27.30
Bicalutamide	46.25	45.41	45.20	51.61
Enzalutamide	31.76	32.27	11.47	53.04

**Table 2:** In vitro antiproliferative activity of the deshydroxy sulfide analogues of bicalutamide (**16**–**18**) across four human prostate cancer cell lines (DU-145, 22Rv, LNCaP, and VCap). All data are mean values from experiments carried out on three separate occasions.

ID	22Rv1 IC_50_ (µM)	DU-145 IC_50_ (µM)	LNCaP IC_50_ (µM)	VCap IC_50_ (µM)
**16**	6.59	8.22	9.44	10.86
**17**	17.36	20.34	9.99	30.85
**18**	>100	>100	83.34	>100
Bicalutamide	46.25	45.41	45.20	51.61
Enzalutamide	31.76	32.27	11.47	53.04

**Table 3:** In vitro antiproliferative activity of the sulfone analogues (**19**–**22**) across four human prostate cancer cell lines (DU-145, 22Rv, LNCaP, and VCap). All data are mean values from experiments carried out on three separate occasions.

ID	22Rv1 IC_50_ (µM)	DU-145 IC_50_ (µM)	LNCaP IC_50_ (µM)	VCap IC_50_ (µM)
**19**	>100	>100	>100	>100
**20**	>100	>100	>100	>100
**21**	24.64	32.89	30.59	43.04
**22**	36.42	39.91	20.49	>100
Bicalutamide	46.25	45.41	45.20	51.61
Enzalutamide	31.76	32.27	11.47	53.04

**Table 4:** In vitro antiproliferative activity of bicalutamide analogues (**27**–**31**) across four human prostate cancer cell lines (DU-145, 22Rv, LNCaP, and VCap). All data are mean values from experiments carried out on three separate occasions.

ID Structure	22Rv1 IC_50_ (µM)	DU-145 IC_50_ (µM)	LNCaP IC_50_ (µM)	VCap IC_50_ (µM)
**27**	7.74	15.03	4.69	8.09
**28**	41.06	59.79	75.61	51.87
**29**	>100	>100	>100	>100
**30**	26.71	34.73	24.01	32.30
**31**	>100	>100	>100	>100
Bicalutamide	46.25	45.41	45.20	51.61
Enzalutamide	31.76	32.27	11.47	53.04

## Data Availability

The data presented in this study are available on request from the corresponding author.

## Sample Availability

Samples of the compounds **10**–**12**, **16**–**18**, **19**–**22**, **27**–**31** are available from the authors.
